# *Babesia* infection in cattle and dogs in Suizhou City, Hubei Province, China

**DOI:** 10.1016/j.imj.2025.100170

**Published:** 2025-02-21

**Authors:** Guandu Wu, Xiaofan Zhou, Fang Guo, Jiao Xu, Jingjing Song, Zhen Jin, Huijie Cao, Ju Tang, Huiya Lu, Zezheng Jiang, Tianmei Yu, Xiaoyong Zhang, Xiaohui Liu, Xue-jie Yu

**Affiliations:** a State Key Laboratory of Virology, School of Public Health, Wuhan University, Wuhan 430071, Hubei Province, China; b Suizhou Center for Disease Control and Prevention, Suizhou 441300, Hubei Province, China; c Suixian County Center for Disease Control and Prevention, Suixian County 441309, Hubei Province, China

**Keywords:** *Babesia*, Cattle, Goats, Dogs, Ticks

## Abstract

**Background:**

Babesiosis is a zoonotic disease caused by the intraerythrocytic parasite *Babesia*, which poses a serious threat to public health. Currently, the prevalence of babesiosis in domestic animals and the genetic diversity of *Babesia* in Central China have not been comprehensively studied.

**Methods:**

In this study, we collected 1093 ticks, including 95.24% (1041/1093) *Haemaphysalis longicornis*, 4.67% (51/1093) *Rhipicephalus microplus,* and 0.09% (1/1093) *Ixodes sinensis*. Blood samples from 216 goats, 56 cattle, and 25 dogs were collected from Suizhou City, Hubei Province, China, and animal blood DNA was extracted for the detection of *Babesia* with PCR.

**Results:**

PCR results showed that 50.00% (28/56) of cattle and 32.00% (8/25) of dogs were *Babesia*-positive, including for *Babesia bovis* 3.57% (2/56), *B. bigemina* 3.57% (2/56), and *B. ovata* 42.86% (24/56) in cattle and *B. gibsoni* 32.00% (8/25) in dogs. All goats (216) and ticks (1093) were *Babesia*-negative.

**Conclusions:**

Our findings showed that *Babesia* infections are prevalent in cattle and dogs in Central China, indicating that babesiosis should be monitored in animals and humans in Central China.

## Highlights

• Our findings show that *Babesia* infection are prevalent in cattle and dogs in central China, indicating that babesiosis should be monitored in in animals and humans in central China.

## Graphical Abstract

**Figure:**
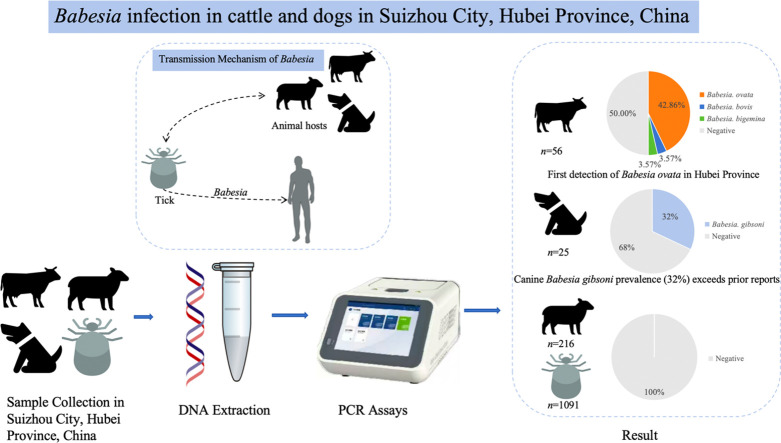

## 1 Introduction

Babesiosis is caused by *Babesia* species, tick-borne protozoan hemoparasites.^1^ *Babesia* species are considered one of the most common blood parasites of mammals, and they infect the erythrocytes of a wide range of vertebrate hosts, including rodents, cattle, dogs, and humans. Since the first description of babesiosis in Romanian cattle, more than 100 *Babesia* species have been identified, some of which are known to infect humans, including *B. microti, B. duncani, B. divergens,* and *B. venatorum.*^2^^,^^3^ Generally, *B. bovis* and *B. bigemina* can cause bovine babesiosis (BB) in cattle, water buffalo, and yaks, leading to enormous economic losses. In China, according to epidemic data from 1947 to the present, the most widespread species in both cattle and water buffalo are *B. bovis* and *B. bigemina.*^4^ A range of minor pathogenic species, including *B. ovata, B. major, B. occultans,* and several unclassified *Babesia* species, are also known to infect cattle.^5^ Tick-borne pathogens are also the main cause of incidence and mortality in pet dogs, and many studies have found that *Babesia* is capable of infecting dogs, including the species *B. canis, B. vogeli, B. rossi, B. gibsoni, B. conradae*, and *B. vulpes,*^6^ and thus are of great public health importance.

Human babesiosis caused by *B. bovis* and *B. bigemina* infection have been reported in Colombia and Ecuador.^7^^,^^8^ Since the reporting of babesiosis in 2011, approximately 1000 cases have been reported annually in the United States, with the predominant prevalent species being *B. microti.*^3^ In a comprehensive review of human babesiosis in Europe, a total of 51 local cases were documented, the majority of which were *B. divergens* infections, with the remainder being *B. microti* and *B. venatorum* infections.^9^ In China, approximately 317 cases were reported from 1943 to 2019, with *B. microti* predominating in South China and *B. venatorum* in North China.^10^ Tick-borne pathogens are often first detected in tick vectors or animal hosts, and subsequently, cases of infection are reported in human populations. Therefore, pathogens found in vectors may be potential human pathogens, and tick-borne disease research cannot be limited to already infected cases. It is also necessary to actively explore the infection capabilities and harmfulness of tick vectors and animal hosts in the environment.

Suizhou City, Hubei Province, is a typical hilly area where animal husbandry is one of the pillar industries. The climate conditions are suitable for the growth and reproduction of ticks, as well as the survival and transmission of various tick-borne pathogens. The *Babesia* infection status in animals and humans in Central China is not clear. The aim of this study was to investigate the prevalence and genetic diversity of *Babesia* in ticks and domesticated animals from rural areas in Suizhou City, Hubei Province.

## 2 Materials and methods

### 2.1 Sample collection and DNA extraction

From the summer of 2023 to the summer of 2024, a total of 1093 ticks and 297 animal blood samples were collected in this study. Ticks were collected from eight areas in Suizhou City (31°19′N to 32°26′N, 112°43′E to 113°46′E) by flagging grasses and removal from animal bodies. Ticks were identified by morphology following a previous study, then representative ticks of each species were confirmed with molecular methods,^11^^,^^12^ including 95.24% (*n* = 1041) *Haemaphysalis longicornis*, 4.67% (*n* = 51) *Rhipicephalus microplus,* and 0.09% (*n* = 1) *Ixodes sinensis*. A triangular spike on the posterior edge of the third segment of the whisker identified *H. longicornis.*^13^^,^^14^ A false head base that was hexagonal in shape, with thick and short tendrils, a shield plate with no eyes or edges, and no toe like protrusions on the inner edge of the first segment of the tendrils characterized *R. microplus.*^15^ *Ixodes sinensis* was characterized by a rectangular base that was slightly wider than its length, and bristles on the ventral surface.^16^ Blood samples were collected from 216 healthy goats, 56 healthy cattle, and 25 dogs (17 healthy dogs and 8 sick dogs) from six areas in Suizhou City. The admission times of these sick pet dogs were concentrated in May 2023 (*n* = 4) and October 2024 (*n* = 4), and the blood collection times for other asymptomatic pet dogs were mainly from June to August 2024. Blood (200 µL) from each animal was subjected to DNA extraction with the Qiagen DNA Kit (Qiagen, Hilden, Germany) following the manufacturer's instructions.

### 2.2 PCR amplification and DNA sequencing

To reduce sample numbers for PCR amplification, the ticks were divided into 233 groups based on their species, gender, life stage, collection location and time, and feeding status.^13^ The weight of each tick group ranged from 20 to 30 mg. Total DNA of animal blood and tick samples was first amplified with *Babesia* universal primers targeting *COX1*. The animal blood and tick samples that were PCR positive for the *COX1* gene were then further amplified with primer pairs for the *cytb* gene and 18S rRNA gene to determine *Babesia* species. All primers used in this study are listed in Table 1. The PCR reactions were performed in a 15-µL mixture containing 7.5 µL 2X Taq Master Mix (Cowin Biotech, Jiangsu Province, China), 1.5 µL of each 10 µM forward and reverse primer (Sangon Biotech, Shanghai, China), 2.5 µL nuclease-free water, and 2 µL sample DNA. Nuclease-free water was used as a negative control in each PCR reaction. The thermocycling conditions of PCR amplifications were 95°C for 5 min, followed by 35 cycles of 95°C for 30 s, annealing at 44°C–53°C for 30 s, 72°C for 30–90 s each cycle, and finally an extension at 72°C for 10 min. The PCR products were analyzed on 1% agarose gels and stained with GoldView II dyes (Solarbio, Beijing, China) and visualized under ultraviolet light. DNA bands with expected sizes were purified with a DNA gel extraction kit (Tsingke, Beijing, China) and were ligated into pMD 19-T vector (Takara Bio, Dalian, Liaoning Province, China) and transformed into *E. coli* DH5α. Both strands of the positive clones were sequenced.

**Table 1:** Primer pairs used for PCR amplification of *Babesia* DNA.

Targeted species	Primers	Primer sequence	Target gene	Size (bp)	Annealing temperature (°C)	References
*Babesia* genus universal primers	Bab-F1	ATWGGATTYTATATGAGTAT	*cox1*	1248	44	^17^
	**Bab-R1**	ATAATCWGGWATYCTCCTTGG				
	**Bab-F2**	TCTCTWCATGGWTTAATTATGATAT		924	49	
	Bab-R2	TAGCTCCAATTGAHARWACAAAGTG				
	18 s F	AGTTTCTGACCTATCAG	18S rRNA	1098	45	^18^
	18 s R	TTGCCTTAAACTTCCTTG				
*B. bovis*	oBbmiF	TGAACAAAGCAGGTATCATAGG	*cytb*	260	50	^19^
	oBbmitR	CCAAGGAGATTGTGATAATTCA				
	**iBbmitF**	TCCACGATCTGTGATACGTCA		195	53	
	**iBbmitR**	CAAATCCTTTGCAAACTCCAA				
*B. bigemina*	oBbigmitF	TCCAACACCAAATCCTCCTA	*cytb*	394	51	^19^
	oBbigmitR	CGTGGGTTTCGTTTTTGTAT				
	**iBbigmitF**	AAGAGATACCATATCAGGGAACCA		250	53	
	**iBbigmitR**	TTGGGCACTTCGTTATTTCC				

*Note*: Bold characters represent nested PCR inner primers.

### 2.3 Sequences analysis

DNA sequences were first analyzed using Seqman Pro (www.dnastar.com) (DNASTAR, Madison, WI, USA).^20^ Qualified sequences were compared with sequences in GenBank with Basic Local Alignment Search Tool (BLAST) (https://blast.ncbi.nlm.nih.gov/Blast.cgi). After removing the primer regions, the sequences from this study, together with the reference sequences from GenBank, were imported into MEGA 7.0 software and aligned with ClustalW. Phylogenetic analysis was performed using the maximum-likelihood method and the Kimura 2-parameter model^21^ with 1000 bootstraps in MEGA 7.0.

## 3 Results

### 3.1 *Babesia* species prevalence in animals

The DNA samples were amplified with *Babesia cox1*, 18S rRNA, and *cytb* gene primers. The DNA sequences of the PCR products were analyzed with BLAST to determine the *Babesia* species. The PCR results showed that the infection rate of *Babesia* in cattle was 50.00% (28/56), including *B. bovis* 3.57% (2/56), *B. bigemina* 3.57% (2/56), and *B. ovata* 42.86% (24/56). The infection rate of *Babesia gibsoni* in dogs was 32.00% (8/25). All goats (0/216) and ticks (0/1093) were *Babesia*–negative (Table 2).

**Table 2:** Prevalence of *Babesia* in cattle, dogs, goats, and ticks in Suizhou, Hubei Province, China 2023–2024.

Animal species	*Babesia* species	Sampling site	Positive rate *n*/N (%)	Total positive rate (%)
Cattle	*B. bovis*	Yindian	3.57 (2/56)	
	*B. bigenima*	Yindian	3.57 (2/56)	
	*B. ovata*	Wanhe	42.86 (24/56)	
	−	Fuhe	0 (0/56)	50
Dog	−	Yindian,Wanhe and Zengdu District	0 (0/25)	
	*B. gibsoni*	Beilekang Pet Hospital	16.00 (4/25)	
	*B. gibsoni*	Baobei Pet Hospital	16.00 (4/25)	32
Goat	−	Yindian, Guangshui, Wudian and Wanhe	0 (0/216)	0
Tick	−	Suizhou City	0 (0/1093)	0

### 3.2 Phylogenetic analysis of *Babesia*

**Fig. 1:**
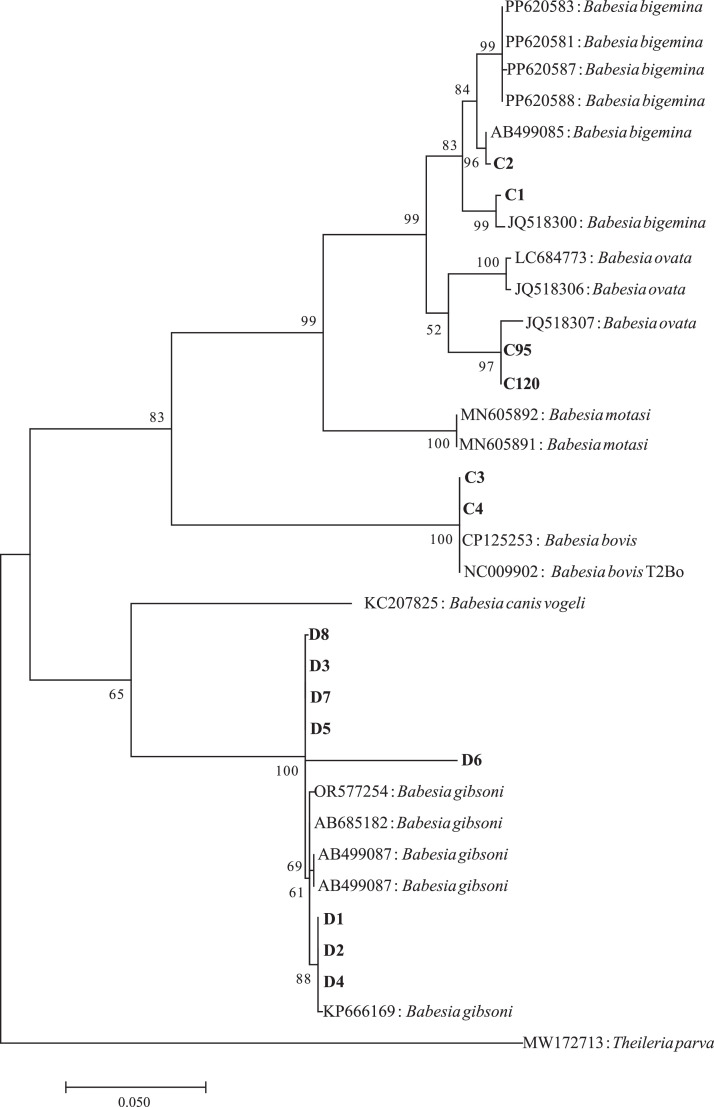
Phylogenetic tree based on the *Babesia cox1* sequence (924 bp). Those from cattle samples are labeled with C, and those from dog samples are labeled with D. The phylogenetic tree was constructed using MEGA 7.0 with the maximum likelihood method of the Kimura 2-parameter model. Only bootstrap values of > 50% are shown. *Theileria parva* was used as an outgroup. The scale bar indicates an evolutionary distance of ten substitutions per site.

Based on the *cox1* gene, BLAST analysis indicated the 36 *Babesia*-positive blood samples contained DNA from 4 *Babesia* species: *B. ovata* (*n* = 24), *B. gibsoni* (*n* = 8), *B. bigenima* (*n* = 2), and *B. bovis* (*n* = 2). Representative strains of each species from this study were used for phylogenetic analysis. Phylogenetic analysis based on the *cox*1 gene showed that *Babesia* sequences clustered together with *B. bovis, B. gibsoni, B. ovata,* and *B. bigemina* (Fig. 1). The *cox*1 sequences of samples C1–C4 were uploaded to GenBank with accession numbers OR700716–OR700716. The two samples (C1 and C2) shared 96%–99% nucleotide identity with *B. bigenima* (AB499085) and *B. bigenima* (JQ518300), and the other two samples (C3 and C4) shared 100% nucleotide identity with *B. bovis* T2Bo (NC009902) and *B. bovis* (CP125253). Phylogenetic analysis indicated that the sequences (represented by C95 and C120) from cattle in this study were most closely related to *B. ovata* (JQ518307) from Lushi County, Henan Province. Phylogenetic analysis showed that the strains from dogs (D1–D8) were in the same clade with *B. gibsoni* (OR577254, AB685182, AB499087, and KP666169). No co-infections were observed in the samples analyzed.

**Fig. 2:**
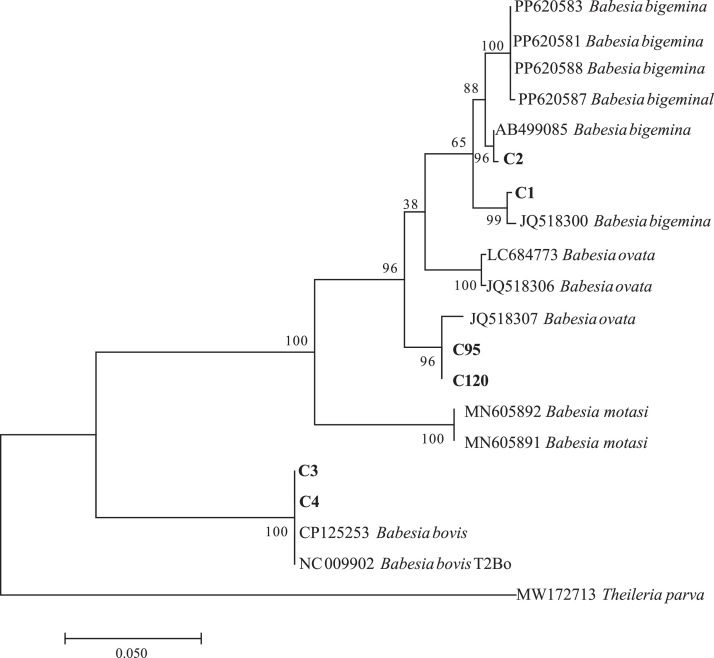
Phylogenetic tree based on the *Babesia cytb* sequence (250 bp). The phylogenetic tree was constructed using MEGA 7.0 with the maximum likelihood method of the Kimura 2-parameter model. Only bootstrap values of > 50% are shown. The scale bar indicates an evolutionary distance of ten substitutions per site.

The phylogeny of the strains from cattle was further analyzed with the *cytb* gene, which indicated that *Babesia* strains from cattle belonged to 3 clusters (Fig. 2). The cattle strains of C1 and C2 were in the same clade as *B. bigemina* (MW939377) from buffalo of Hainan Province, China. The strains of C3 and C4 were in the same clade and shared 100% nucleotide identity with *Babesia bovis* (AB454079) and *B. bovis* T2Bo (NC009902). Phylogenetic analysis with *cytb* sequences showed that the strains from cattle, including C95 and C120, were in the same clade as *B. ovata* (JQ518307).

**Fig. 3:**
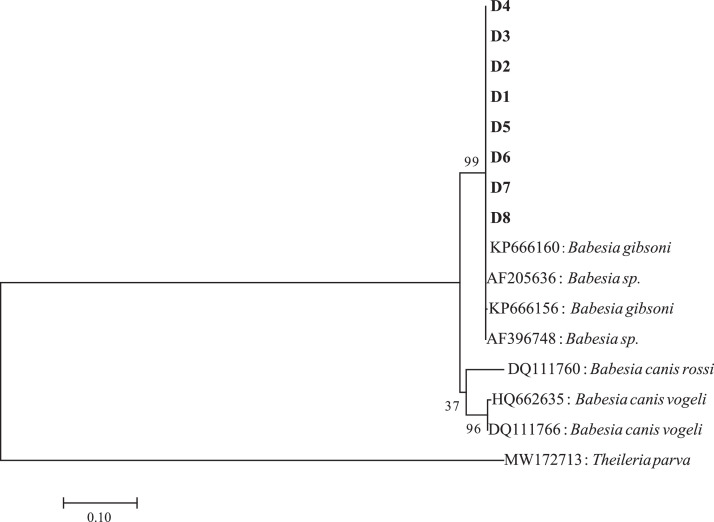
Phylogenetic tree based on the *Babesia* 18S rRNA sequence (1098 bp). The phylogenetic tree was constructed using MEGA 7.0 with the maximum likelihood method of the Kimura 2-parameter model. The scale bar indicates an evolutionary distance of ten substitutions per site.

The phylogeny of these strains from dogs were further analyzed with the 18S rRNA gene, which indicated that *Babesia* strains from dogs belonged to one cluster (Fig. 3). Phylogenetic analysis with 18S rRNA sequences showed that the strains from dogs, including D1–D8, were in the same clade as *B. gibsoni* (KP166156 and KP166160) from dogs of Wuhan City, Hubei Province, China.

## 4 Discussion

The animal samples used for this study were collected in Suizhou City, located in the northern part of Hubei Province in Central China. The region has mild weather and four distinct seasons, contributing to its diverse flora and fauna. Areas at a low altitude and with rich vegetation are more suitable for tick growth and reproduction, and thus the incidence of tick-borne parasites.^22^, ^23^, ^24^ Domesticated animals in Suizhou are mostly raised in free-range grazing conditions, which makes them more likely to be exposed to ticks, and also increases the chances of pathogen transmission.^25^

In this study, *B. ovata, B. bovis*, and *B. bigemina* were found in the blood of healthy cattle, and *B. gibsoni* was found in sick dogs from a pet hospital in Suizhou City, Hubei Province. The *Babesia* species identified varied from place to place, possibly because of the small sample sizes. *Babesia bovis* and *B. bigemina* have been found in buffalo in Hubei Province before,^26^ and *B. gibsoni* has been found in dogs in Wuhan, Hubei Province,^27^ but *B. ovata* has not been found previously in Hubei Province.^4^ *Babesia ovata* has been confirmed to exist in Gansu, Henan, and Sichuan provinces,^28^ and this study was the first to discover *Babesia ovata* in Hubei Province, expanding our understanding of its geographical distribution in China. We also conducted testing on tick samples from Suizhou City, and we did not find *B. bovis* or *B. bigemina* infections. This may be due to the fact that most of the tick samples we collected were *H. longicornis* (95.2%, 1041/1093), and the main vector of transmission for *B. bigemina* and *B. bovis* is *R. microplus.*^4^ Recently, researchers have detected *B. bigemina* in *R. microplus* in Yingshan County of Hubei Province, but not in *H. longicornis,* supporting this conclusion.^29^ While *B. ovata* and *B. gibsoni* are also transmitted by *H. longicornis,* we did not find *B. ovata* or *B. gibsoni* infections.^30^ The reason for this may stem from various innate immune mechanisms that shield ticks from microbial invasion, thereby resulting in a shorter duration of *Babesia* spp. infection.^31^ Another possible reason could be the involvement of other ticks in the transmission of *B. ovata.*^5^

*Babesia bovis* and *B. bigemina* are recognized as the primary causative agents of bovine babesiosis in cattle and related ruminants.^4^ *Babesia bigemina* infections typically manifest with low parasitemia levels, whereas *B. bovis* induces significantly higher parasitemia, leading to more severe clinical outcomes, and these two species of *Babesia* are considered to have the greatest economic impact on cattle health.^32^ *Babesia ovata* were thought to have low pathogenicity in cattle; however, in cases of weakened immune function, parasites can rapidly multiply, resulting in hyperparasitemia, which can further lead to severe anemia and the development of anemia-related clinical syndromes.^33^ *Babesia bovis* and *B. bigemina* DNA has been detected in bovine blood samples from Indonesia, Malaysia, Myanmar, the Philippines, Thailand, and Vietnam, with detection rates as high as 61.70% (cPCR) for *B. bigemina* and 50.70% (nPCR) for *B. bovis.*^34^ Epidemiological studies showed that the infection rates of *B. bigemina* and *B. bovis* were as high as 90% in some locations of China, especially in Hunan, Hubei, Yunnan, Guizhou, and Sichuan provinces.^26^ In contrast, the infection rate of *B. ovata* in cattle is low in China,^32^ and the detection rate of DNA samples extracted from cattle blood collected in Thailand was reported to be 3.42%.^35^ In Vietnam, the detection rate was 1.20%.^36^ *Babesia bovis* and *B. bigemina* were also the main species detected in cattle herds in Bangladesh, with very few reports of *B. ovata.*^37^ In this study, the infection rate of *B. bigemina* or *B. bovis* in cattle blood samples (*n* = 4) collected in 2023 was 100%, but it was 0% (0/52) in the samples collected in 2024. Differences in infection rates between years may be caused by collection sites and times. The detection rate of *B. ovata* was as high as 46.15% in cattle blood samples (*n* = 52) collected in 2024, indicating that cattle play an important role in the transmission cycle of *Babesia* in Suizhou City. The observed discrepancies between our findings and previous studies may be attributed to multiple factors: methodological variations in pathogen detection,^38^ the transient parasitemia dynamics of *Babesia* spp. in hosts,^31^ and geographic heterogeneity in dominant tick vectors and potential sampling biases.

*Babesia gibsoni* was the predominant pathogen causing babesiosis in dogs in the central, southern, and eastern regions of China.^39^ Molecular epidemiological studies have reported a *B. gibsoni* infection rate of 28.6% in domestic dogs in India, while stray dog populations in Bangladesh exhibited a slightly higher prevalence of 30%.^37^^,^^40^ Previous studies have shown that the infection rates of *B. gibsoni* in dogs in Wuhan, Hubei Province, Shaanxi Province, Hong Kong Special Administrative Region (China), and Anhui Province are 11.9%, 7.2%, 3.7%, and 2.2%, respectively^41^^,^^42^; our study recorded 32.00% (8/25) in Suizhou City, significantly higher than the aforementioned regions in China. The blood samples that tested positive were collected from 8 sick dogs from different pet hospitals in the same neighborhood in 2023 and 2024, indicating that the infection of local dogs by *B. gibsoni* cannot be ignored. All 8 sick dogs had serious clinical symptoms, such as pyrexia, hemoglobinuria, anorexia, shock, jaundice, and anemia. The blood samples (*n* = 17) from healthy dogs were negative for *Babesia*. In previous studies, a significant proportion of *B. gibsoni*-infected dogs were subclinical^43^; weight loss was a potential feature, with most experiencing lethargy, anorexia, and anemia, and a small portion experiencing diarrhea and vomiting.^30^ The symptoms of the sick dogs included in this study were similar to those described in the previous reports. Therefore, the differences in infection rates may be due to regional and temporal factors, or a lack of clinical symptoms leading to the underrepresentation of cases. With *B. gibsoni*, as tick-transmitted parasites, the prevalence of piroplasmosis depends on the distribution of the tick vectors. Dog bites, blood transfusions, and transplacental transmission may represent alternative routes of transmission, and regardless of whether significant clinic manifestations are present, infected dogs can become reservoirs and infect other *B. gibsoni*-free dogs.^27^^,^^44^ There are a large number of people and dogs in China,^45^ and there is a daily surge in pet ownership, necessitating the urgent clarification of the epidemiology of zoonotic infections transmitted from dogs to prevent the spread of pathogens.

Since the first case of human babesiosis was reported in China in 1944, over 314 cases of human babesiosis or asymptomatic cases had been reported in 14 provinces in China as of 2019, including Xinjiang Uygur Autonomous Region, Chongqing City, Henan Province, Yunnan Province and Guangxi Zhuang Autonomous Region. Species causing human babesiosis include *B. crassa, B. diversens, B. microti, B. venatorum,* and *B. crassa-*like species.^10^ In addition, *B. bovis* has been found in human blood in Colombia, with an infection rate of 6%.^8^ Similarly, *B. bigemina* was first discovered in a patient in the Amazon basin,^7^ indicating the potential for the zoonotic transmission of *B. bovis* and *B. bigemina*. Our study identified *B. bovis, B. bigemina*, and *B. ovata* in cattle from Suizhou City, Hubei Province, suggesting that the risk of human and animal babesiosis in Central China may be underestimated owing to limited surveillance. Suizhou City as a hilly agricultural region with intensive livestock farming and a climate highly conducive to tick proliferation, thus it is necessary to strengthen the surveillance of tick borne pathogens to reduce the risk of zoonotic diseases.

A key limitation of this study was the lack of serological assessment of host antibody levels. A TaqMan polymerase chain reaction (TaqMan PCR) method was found to be a promising diagnostic method with high sensitivity and specificity for detecting parasites,^46^ but this method has certain limitations. Because of the host's own immune mechanisms, sometimes pathogens exist in the host's body for a short period of time and cannot be detected by PCR.^31^ Enzyme-linked immunosorbent assay (ELISA) is easy to perform, inexpensive and reproducible.^38^ Serological assays exhibit higher sensitivity than PCR for detecting certain pathogens.^41^ A previous study showed that blood containing an extremely low *B. microti* density can still stimulate the host to produce high concentrations of antibodies, and this high antibody level is maintained for a long period (up to 270 days post infection), but the nested PCR test is limited by a low density of parasites in the blood.^47^ In summary, ELISA has better sensitivity than PCR for parasites at a low density level, and antibodies exist for a longer period of time. If the two methods are combined, the experimental results obtained will have higher credibility.

## Funding

This study was supported by the 10.13039/501100001809National Natural Science Foundation of China (grant number 32470155) and Key R&D Program of Hubei Province, China (2022BCE063).

## CRediT authorship contribution statement

**Guandu Wu:** Writing – original draft, Methodology, Investigation, Formal analysis, Data curation. **Xiaofan Zhou:** Writing – original draft, Investigation, Conceptualization. **Fang Guo:** Resources, Investigation. **Jiao Xu:** Investigation. **Jingjing Song:** Resources, Investigation. **Zhen Jin:** Resources. **Huijie Cao:** Resources. **Ju Tang:** Investigation. **Huiya Lu:** Investigation. **Zezheng Jiang:** Investigation. **Tianmei Yu:** Investigation. **Xiaoyong Zhang:** Resources. **Xiaohui Liu:** Resources. **Xue-jie Yu:** Writing – review & editing, Supervision, Data curation, Conceptualization.

## Appendix Supplementary materials

Image, application 1
