# Supplementary material for: Babesia infection in cattle and dogs in Suizhou City, Hubei Province, China
Source: Infect Med (Beijing). 2025 Feb 21;4(1):100170. doi: 10.1016/j.imj.2025.100170 (PMC11930583; doi:10.1016/j.imj.2025.100170)
Supplement: Supplementary file 1 [file mmc1.docx]

Supplemental Table: Sampling location information

| Sampling Sites | Sample Types | Longitude (E) | Latitude (N) |
| --- | --- | --- | --- |
| Wanhe Town | tick, dog blood, cattle blood, goat blood | 113.28 | 32.23 |
| Suizhou County | tick | 113.51 | 32.13 |
| Yindian Town | tick, dog blood, cattle blood, goat blood | 113.56 | 32.05 |
| Guangshui City | goat blood | 113.80 | 31.68 |
| Zengdu District | tick, dog blood | 113.43 | 31.89 |
| Fuhe Town | tick, cattle blood | 113.57 | 31.52 |
| Wushengguan Town | tick | 114.03 | 31.64 |
| Huantan Town | tick | 112.97 | 31.72 |
| Caihe Town | tick | 113.83 | 31.72 |
| Wudian Town | goat blood | 113.65 | 31.93 |
